# Comparison of the Clinical Characteristics of Pneumocystis Pneumonia between Patients with Rheumatoid Arthritis Being Treated with Biologics and Those Being Treated without Biologics

**DOI:** 10.1155/2017/3710652

**Published:** 2017-07-12

**Authors:** Mitsuhiro Akiyama, Yuko Kaneko, Tsutomu Takeuchi

**Affiliations:** Division of Rheumatology, Department of Internal Medicine, Keio University School of Medicine, Tokyo, Japan

## Abstract

**Objective:**

The aim of this study was to compare the clinical characteristics of pneumocystis pneumonia (PCP) between patients with rheumatoid arthritis (RA) being treated with biologics and those being treated without biologics.

**Methods:**

From 8,630 patients with RA in our institution, we enrolled 24 patients who had developed PCP during the course of their treatment. They were divided into two groups according to the treatment they were receiving for RA: the biologics group (*n* = 12) and the nonbiologics group (*n* = 12). Clinical characteristics of PCP were compared between the two groups.

**Results:**

At PCP diagnosis, the biologics group showed significantly lower serum levels of *β*-D-glucan and C-reactive protein than the nonbiologics group, while the biologics group had significantly higher lymphocyte counts than the nonbiologics group. In the nonbiologics group, lower lymphocyte counts were associated with higher *β*-D-glucan levels; however, this was not observed in the biologics group.

**Conclusion:**

The finding that RA patients being treated with biologics developed PCP with relatively normal lymphocyte counts and lower *β*-D-glucan levels suggests that the pathophysiology of PCP in those patients is different from that in patients being treated with other antirheumatic drugs.

## 1. Introduction

Pneumocystis pneumonia (PCP) is a rare but critical complication in immunosuppressed patients [1]. It was initially discovered in human immunodeficiency virus (HIV) positive patients in the 1980s [2]. In the following years, PCP cases increased in patients with rheumatoid arthritis (RA) being treated with disease-modifying antirheumatic drugs (DMARDs), with high mortality rates recorded [3, 4]. More recently, PCP cases have also been reported during treatment with biologic agents targeted at specific molecules [5, 6].

While immunosuppressive therapy is identified as a risk factor for the development of PCP, through lymphocyte count suppression [7], a previous study reported that PCP could develop during treatment with biologic agents in patients with RA, without apparent lymphocytopenia [8]. This suggests that distinct mechanisms may be involved in the development of PCP during treatment with biologic agents. However, little is known about the differences in the clinical characteristics of PCP during treatment with biologic agents in comparison with nonbiologic DMARDs.

The objective of this study was to elucidate the differences in the clinical characteristics of PCP between patients being treated with biologic agents and patients being treated with nonbiologic DMARDs.

## 2. Patients and Methods

### 2.1. Study Design

The study was a retrospective, observational clinical study conducted at our institution with approval by the institutional ethics committee. Written informed consent was waived based on Japanese guidelines.

From a total of 8,630 consecutive patients with RA who visited our institution between March 1999 and September 2016, all patients who were diagnosed with PCP were enrolled in the analysis.

PCP was diagnosed as definite when patients met all the following criteria [9]: (a) clinical manifestations and findings compatible with PCP on chest computed tomography (CT), (b) microscopic detection of* Pneumocystis jirovecii* or positive polymerase chain reaction test results for* Pneumocystis jirovecii* DNA in respiratory specimens, (c) increased *β*-D-glucan serum levels. PCP was diagnosed as probable when patients met criterion (a), with either criterion (b) or (c) in addition [9]. The manufacturer suggested that the upper normal limit for serum *β*-D-glucan was 11.3 pg/mL (Wako Pure Chemical Industries, Tokyo, Japan), but we defined 31.1 pg/mL as a cut-off value in this study to exclude* Pneumocystis jirovecii* colonization, based on a previous study [10].

### 2.2. Clinical Assessments

Demographic characteristics (age, sex, smoking status, and duration from onset of symptoms to PCP diagnosis), comorbidities, associated medical conditions, and laboratory findings (white blood cell count, lymphocyte count, lactate dehydrogenase [LDH], C-reactive protein [CRP], immunoglobulin G [IgG], Krebs von den Lungen-6 [KL-6], and *β*-D-glucan) were retrospectively reviewed. The severity of pneumonia was evaluated with the oxygenation index determined from the arterial oxygen tension and the fraction of inspired oxygen (PaO_2_/FiO_2_ ratio).

### 2.3. Statistical Analysis

Continuous variables were expressed as mean ± standard error. Mann–Whitney *U* test and Fisher's exact test were used to compare continuous and categorical variables, respectively. The relationships between variables were analysed by Spearman correlation coefficient. SPSS version 22.0 (IBM, Armonk, NY, USA) was used for all statistical analyses, and *P* < 0.05 was considered as significant.

## 3. Results

### 3.1. Recruitment of Patients

**Figure 1:**
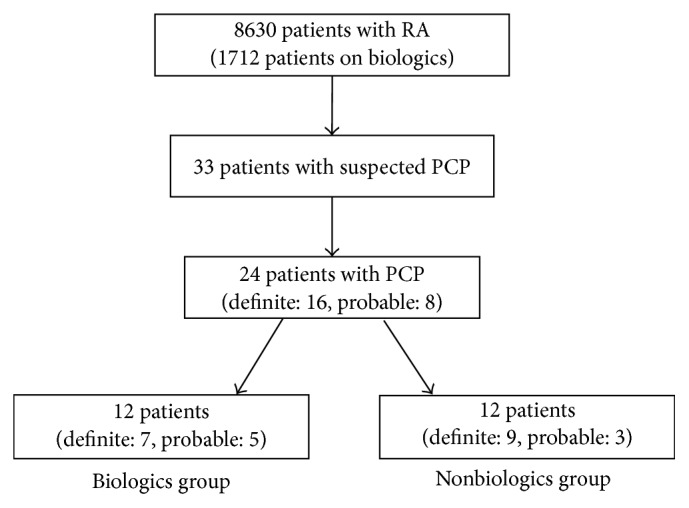
Flowchart of inclusion of patients. RA, rheumatoid arthritis. PCP, pneumocystis pneumonia.

Figure 1 is an outline showing how patients were recruited for this study. Of the 8,630 patients with RA, 1712 patients received biologics. Thirty-three patients were suspected to have PCP. Of these 33 patients, 9 patients were excluded from the analysis because they did not satisfy the diagnostic criteria stated above. Consequently, a total of 24 patients were enrolled in this study (definite PCP, *n* = 16, and probable PCP, *n* = 8) and divided into two groups according to their treatment for RA (the biologics group, *n* = 12 (definite, *n* = 7 and probable, *n* = 5), and the nonbiologics group, *n* = 12 (definite, *n* = 9 and probable, *n* = 3)). Patients in the biologics group received infliximab, *n* = 7; adalimumab, *n* = 2; etanercept, *n* = 1; golimumab, *n* = 1; and abatacept, *n* = 1.

### 3.2. Comparison between Patients Being Treated with Biologics and Those Being Treated with Nonbiologics

Baseline demographic characteristics of the patients at the time of their diagnosis of PCP are shown in Table 1. The patients in the nonbiologics group were significantly older than those in the biologics group (72.5 versus 66.4 years, *P* = 0.045), but no significant differences were found in smoking history (33.3% versus 41.7%,* P* = 1.00), duration from onset of symptoms to PCP diagnosis (7.7 versus 11.1 days, *P* = 0.44), comorbidities, and concomitant immunosuppressive treatments, between the biologics and nonbiologics groups.

While the biologics group had significantly lower serum levels of *β*-D-glucan (100 versus 231 pg/mL, *P* = 0.039) and CRP (5.2 versus 10.6 mg/dL, *P* = 0.039) than the nonbiologics group, the biologics group had significantly higher lymphocyte counts than the nonbiologics group (1429 versus 585 cells/*μ*L, *P* = 0.033), implying that patients on biologics develop PCP with almost normal lymphocyte counts accompanied with low *β*-D-glucan levels. No significant differences were found in total white blood cell counts (5358 versus 7217 cells/*μ*L, *P* = 0.51), the levels of IgG (1127 versus 896 cells/*μ*L, *P* = 0.26), LDH (298 versus 389 U/L, *P* = 0.11), KL-6 (632 versus 1008 U/mL, *P* = 0.22), and the PaO_2_/FiO_2_ ratio (293 versus 281, *P* = 0.53). The survival rate was not different in both groups (91.7% versus 83.3%,* P* = 1.00).

### 3.3. Factors Associated with the Severity of PCP

**Figure 2:**
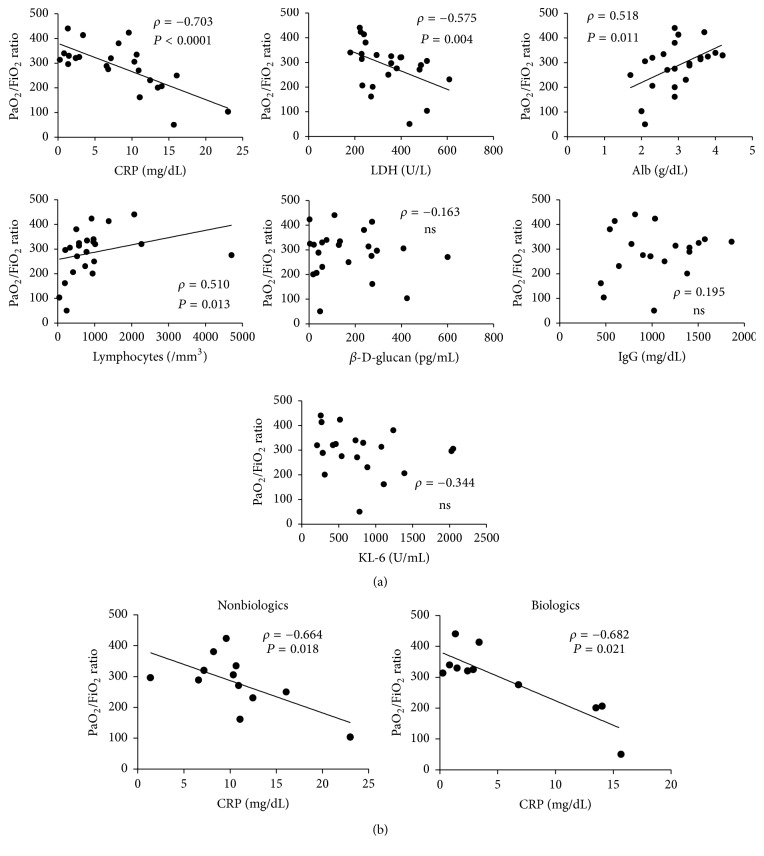
The association of PaO_2_/FiO_2_ ratio with laboratory findings. (a) Total PCP patients. (b) The biologics group and the nonbiologics group. CRP, C-reactive protein. LDH, lactate dehydrogenase. KL-6, Krebs von den Lungen-6. NS, not significant.

Furthermore, we analysed the relationship between the PaO_2_/FiO_2_ ratio, which is used to assess the severity of pneumonitis, and laboratory findings. In all the patients (*n* = 24), serum levels of CRP and LDH negatively correlated with the PaO_2_/FiO_2_ ratio, while lymphocyte counts and serum albumin levels positively correlated with the PaO_2_/FiO_2_ ratio (Figure 2(a)). However, when we performed the same analysis separately in the biologics group and in the nonbiologics group, only the CRP levels negatively correlated with the PaO_2_/FiO_2_ ratio in both groups (Figure 2(b)). The significant correlation with the other variables, except for LDH in the nonbiologics group, was no longer detected in either group, suggesting that the CRP level was the most important index for assessing the severity of PCP.

### 3.4. The Association of Lymphocyte Count with *β*-D-Glucan Levels

**Figure 3:**
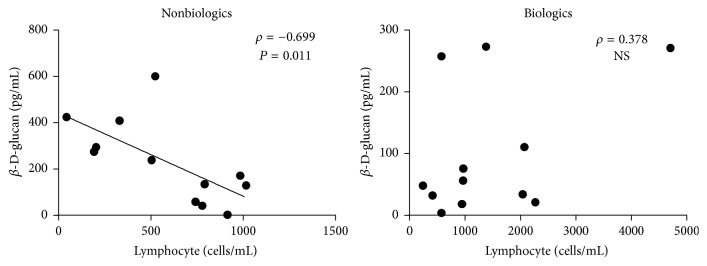
The association of lymphocyte counts with serum levels of *β*-D-glucan. NS, not significant.

We analysed the relationship between the three parameters that differed between the biologics and nonbiologics groups (lymphocyte count, *β*-D-glucan, and CRP). In the nonbiologics group, lower lymphocyte counts were associated with higher *β*-D-glucan levels (Figure 3), implying that the compromised immune system of patients in the nonbiologics group was related to the growth of* Pneumocystis jirovecii*. Interestingly, this relationship was not found in the biologics group (Figure 3). CRP levels were not related to either lymphocyte counts or *β*-D-glucan levels in either group.

Further on, we performed the same analysis on the 16 patients with definite PCP (the biologics group, *n* = 7; the nonbiologics group, *n* = 9). Likewise, lower lymphocyte counts were associated with higher *β*-D-glucan levels only in the nonbiologics group (*ρ* = −0.683, *P* = 0.042), while this association was not observed in the biologics group (*ρ* = 0.679, *P* = 0.094).

## 4. Discussion

In this study, we revealed that, in patients with RA who are being treated with biologic agents, PCP developed with almost normal lymphocyte counts and lower *β*-D-glucan levels, whereas in those being treated with nonbiologic DMARDs, PCP was marked by high *β*-D-glucan levels with apparent lymphocytopenia. The findings suggest that the pathophysiology of PCP in patients being treated with biologic agents is different from that in patients being treated with nonbiologic DMARDs. Furthermore, we found that only serum CRP levels, but not *β*-D-glucan levels, reflected the severity of PCP in patients with RA.

Assuming that *β*-D-glucan levels indicate the growth of* Pneumocystis jirovecii* and lymphocyte counts represent the degree of compromise on the immune system, the differences in the burden of* Pneumocystis jirovecii* and immunosuppression in our study suggest that the mechanism of PCP development with the use of biologic agents is substantially different from that with the use of nonbiologic DMARDs. A previous study reported that alveolar macrophages are crucial in mediating the clearance of* Pneumocystis jirovecii* from the lungs [11], through the production of a large amount of tumour necrosis factor-*α* (TNF-*α*) [12]. An experiment on murine models of PCP proved that the clearance of* Pneumocystis jirovecii* infection was impaired when TNF-*α* was neutralized by TNF-*α* inhibitors [13]. Thus, PCP development during the use of biologic agents, which directly inhibit cytokines such as TNF-*α*, may be caused by impaired primary host immune responses of alveolar macrophages, rather than by lymphocyte suppression. Conversely, the pathogenesis of PCP during the use of nonbiologic DMARDs may be ascribed to immunosuppression that manifests with lymphocytopenia. In fact, lymphocytes play a crucial role in host defence against* Pneumocystis jirovecii*, both in humans and in murine models, with an increased risk of PCP development in patients with markedly decreased lymphocyte counts [14, 15]. Consistent with these reports, our study showed that there is a significant correlation between lower lymphocyte counts and the burden of* Pneumocystis jirovecii,* indicated by serum *β*-D-glucan levels in patients receiving nonbiologic DMARDs.

Respiratory impairment is more closely associated with the extent of lung inflammation than with the amount of* Pneumocystis jirovecii* burden in HIV-infected patients, and the same finding was reported in non-HIV-infected patients [16]. Similarly, our present study showed that respiratory impairment, represented by the PaO_2_/FiO_2_ ratio, was related to the levels of serum CRP, but not to the levels of serum *β*-D-glucan in both the biologics and the nonbiologics groups. PCP severity may be a result of lung inflammation, disturbing gas exchange, and may be unaffected by the quantity of* Pneumocystis jirovecii*.

We note that our study has limitations. This is a retrospective, observational study with a small sample size, although the initial sample of patients with RA, with or without PCP, was of a large number. Also, there is a little possibility that some lost-to-follow-up patients may have developed PCP; however, we believe they would be very few considering the low incidence of PCP and would not change the results of our study. We believe that our present study is important for increasing the understanding of this rare, critical complication of PCP in patients with RA being treated with biologic agents. Further prospective studies in larger cohorts of patients are needed to confirm our results.

In conclusion, in patients with RA being treated with biologic agents, PCP develops with nearly normal lymphocyte counts and low *β*-D-glucan levels. Rheumatologists should recognize these newly identified characteristics of this rare but critical complication during treatment with biologic agents to ensure early diagnosis and appropriate treatment.

## Figures and Tables

**Table 1:** Comparison of demographic characteristics of RA-PCP at diagnosis.

Characteristics	All patients *N* = 24	Biologics group *N* = 12	Nonbiologics group *N* = 12	*P*value^*∗*^
Age, years, mean (SEM)	69.5 (1.5)	66.4 (2.2)	72.5 (1.7)	0.045
Female, *n* (%)	20/24 (83.3)	10/12 (83.3)	10/12 (83.3)	1.00
Smoking, *n* (%)	9/24 (56.3)	4/12 (33.3)	5/12 (41.7)	1.00
Disease duration, days, mean (SEM)	9.4 (2.2)	7.7 (1.0)	11.1 (4.2)	0.44
Comorbidity, *n* (%)				
Renal dysfunction^‡^	13/24 (54.2)	6/12 (50.0)	7/12 (58.3)	1.00
Lung disease^‡‡^	10/24 (41.7)	4/12 (33.3)	6/12 (50.0)	0.68
Heart failure	3/24 (12.5)	1/12 (8.3)	2/12 (16.7)	1.00
Liver dysfunction^‡‡‡^	10/24 (41.7)	4/12 (33.3)	6/12 (50.0)	0.68
Diabetes mellitus	7/24 (29.2)	3/12 (25.0)	4/12 (33.3)	0.67
Concomitant treatment, *n* (%)				
Glucocorticoid	17/24 (70.8)	7/12 (58.3)	10/12 (83.3)	0.37
Dose of glucocorticoid (mg/day), mean (SEM)	7.7 (1.7)	5.7 (1.1)	9.1 (2.7)	0.60
Methotrexate	23/24 (95.8)	11/12 (91.7)	12/12 (100.0)	1.00
Dose of methotrexate (mg/week), mean (SEM)	9.2 (0.6)	8.9 (0.6)	9.5 (0.9)	0.93
Laboratory findings				
PaO_2_/FiO_2_ ratio, mean (SEM)	287 (20)	293 (33)	281 (25)	0.53
White blood cells (/*μ*L), mean (SEM)	6288 (700)	5358 (732)	7217 (1165)	0.51
Lymphocytes (/*μ*L), mean (SEM)	1007 (201)	1429 (357)	585 (96)	0.033
Albumin, g/dL, mean (SEM)	3.0 (0.1)	3.3 (0.2)	2.7 (0.2)	0.06
CRP, mg/dL, mean (SEM)	7.9 (1.2)	5.2 (1.7)	10.6 (1.5)	0.039
IgG, mg/dL, mean (SEM)	1023 (91)	1127 (124)	896 (128)	0.26
LDH, U/L, mean (SEM)	343 (24)	298 (24)	389 (38)	0.11
KL-6, U/mL, mean (SEM)	793 (117)	632 (100)	1008 (226)	0.22
*β*-D glucan, pg/mL, mean (SEM)	166 (32)	100 (30)	231 (52)	0.039
Death, *n* (%)	3/24 (12.5)	1/12 (8.3)	2/12 (16.7)	1.00

^*∗*^Comparison between biologics and nonbiologics group. ^‡^Renal dysfunction: estimated glomerular filtration rate under 60 mL/min/1.73 m^2^. ^‡‡^Lung disease: interstitial lung disease, bronchiolitis, and chronic obstructive pulmonary disease. ^‡‡‡^Liver dysfunction: alanine aminotransferase over 37 IU/L. Biologic agents: infliximab, etanercept, adalimumab, golimumab, and abatacept. RA, rheumatoid arthritis; PCP, pneumocystis pneumonia; LDH, lactate dehydrogenase; CRP, C-reactive protein; KL-6, Krebs von den Lungen-6.
